# The social transmission of stress in animal collectives

**DOI:** 10.1098/rspb.2021.2158

**Published:** 2022-05-11

**Authors:** Hanja B. Brandl, Jens C. Pruessner, Damien R. Farine

**Affiliations:** ^1^ Centre for the Advanced Study of Collective Behaviour, University of Konstanz, 78457 Konstanz, Germany; ^2^ Department of Psychology, University of Konstanz, 78457 Konstanz, Germany; ^3^ Department of Collective Behaviour, Max Planck Institute of Animal Behavior, 78457 Konstanz, Germany; ^4^ Department of Evolutionary Biology and Environmental Studies, University of Zurich, 8057 Zurich, Switzerland; ^5^ Division of Ecology and Evolution, Research School of Biology, Australian National University, Canberra, ACT 2600, Australia

**Keywords:** behavioural endocrinology, collective behaviour, hormonal coregulation, physiological contagion, social behaviour, stress contagion

## Abstract

The stress systems are powerful mediators between the organism's systemic dynamic equilibrium and changes in its environment beyond the level of anticipated fluctuations. Over- or under-activation of the stress systems' responses can impact an animal's health, survival and reproductive success. While physiological stress responses and their influence on behaviour and performance are well understood at the individual level, it remains largely unknown whether—and how—stressed individuals can affect the stress systems of other group members, and consequently their collective behaviour. Stressed individuals could directly signal the presence of a stressor (e.g. via an alarm call or pheromones), or an acute or chronic activation of the stress systems could be perceived by others (as an indirect cue) and spread via social contagion. Such social transmission of stress responses could then amplify the effects of stressors by impacting social interactions, social dynamics and the collective performance of groups. As the neuroendocrine pathways of the stress response are highly conserved among vertebrates, transmission of physiological stress states could be more widespread among non-human animals than previously thought. We therefore suggest that identifying the extent to which stress transmission modulates animal collectives represents an important research avenue.

## 1. Introduction

The activation of stress systems in response to threats in its environment is a fundamental adaptive mechanism that maintains the dynamic equilibrium of an organism. When the brain perceives and identifies a stressor—broadly defined as an environmental stimulus that is perceived as threatening and could push the homeostasis of the body's internal systems beyond the limits of their dynamic physiological thresholds—the body instigates autonomic, endocrine and behavioural changes, together referred to as the stress response (reviewed in [1]). The stress response arises through various stress systems, including the parasympathetic and the sympathetic branch of the autonomic nervous system (fight or flight response), and the endocrine system with its hypothalamic–pituitary–adrenal/interrenal axis (e.g. glucocorticoid-mediated metabolic changes). These stress-related changes, typically comprising psychological and behavioural adjustments, are often summarily referred to as ‘stress’ (and result in individuals being ‘stressed’). Overall, stress responses allow animals to adapt to stressors (see [2–4] for more detailed reviews), but can also be costly (box 1), particularly when stress becomes chronic (box 2). While a stress response is inherently a within-individual process, it can also be mediated by the social environment.

Box 1. **Balancing costs and benefits—trade-offs of the stress response.**

Despite the vital function of the stress response helping animals cope with and survive physical or social stressors, negative consequences have been linked to acute [5,6] and chronic activation of the stress system (e.g. [4,7–10]). One explanation for the apparent conundrum of the survival-supporting stress response causing harm to organisms is that, as with most hormones, glucocorticoids—to which harmful effects are often attributed—have pleiotropic effects [11]. Glucocorticoids can induce a wide range of activating, modulating and attenuating responses (reviewed in [2]), simultaneously affecting different tissues and suites of traits. We can thus expect trade-offs between different hormonal actions, where not all mediated traits necessarily lead to a fitness increase [11,12]. The optima for hormone excretion under acute or chronic stress might differ [13] and short-term benefits might need to be balanced against costs of chronic glucocorticoid exposure that can increase the risk for diseases through ‘wear and tear’ [7] (see box 2). With psychological or psychosocial stressors in humans and non-human primates, the stress response itself can be maladaptive [14] or mismatches occur between animals and their response to anthropogenic stressors [15], potentially leading to costs without benefits. Traditionally, these costs and benefits are measured at an individual level. Thus, including group-level outcomes in the equation may represent a missing component for understanding net fitness benefits of stress responses in group living animals.

Box 2. **Coping with repeated and chronic stress exposure.**

In wild animals, chronic or repeated stress can stem from anthropogenic noise [16], light pollution [17], translocation [18] or persistent [8] effects of predation pressure [8]. Repeated exposure to acute stressors can change physiological set points and lower the threshold at which physiological mediators start to act pathologically (see homeostatic overload [4]), paralleling effects of chronic stress and making individuals more vulnerable to other prospective stressors [19]. Repeated capture stress makes little penguins (*Eudyptula minor*) react more strongly to subsequent stressors [20] and penguins from a high-disturbance colony give stronger heart rate responses to predator cues [21]. Further, evidence is accumulating that not only the ability to trigger an appropriate stress response is an essential survival mechanism, but also the ability to switch off the stress response when the threat has passed (e.g. [22,23]). Compromised negative feedback of the stress system has been correlated with surviving famine events in Galápagos marine iguanas [23]. Accordingly, individual phenotypes and their flexibility regarding stress responsiveness are determining factors for how well individuals cope with challenges, but a group's composition in terms of stress response phenotypes and different physiological setpoints may be additionally underpinning how and how well individuals in a social group cope.

Most animals spend at least part of their lives associating socially with others [24]. Sociality can mediate the stress responses in multiple ways, which should be considered when evaluating the impact of stressors on animals. Being in a group can inherently alter the perception and processing of environmental stressors, playing a role in determining what response (if any) is triggered. For example, mice exposed to a novel environment alone showed stronger immuno-endocrine responses to this mild stressor compared to group-housed mice [25]. Social interactions, such as cooperative actions or agonistic fights, can also stimulate stress responses (reviewed in [26]). For example, greylag geese (*Anser anser*) responded to agonistic interactions with heart rate increases, whereby more intense interactions or being attacked by a frequently winning opponent led to stronger responses [27]. Another potentially impactful social process is stress transmission (sometimes also referred to as *stress contagion* or *physiological resonance*), where a stress response system becomes activated after interacting with another stressed individual (e.g. [28]).

Stress transmission occurs when a previously stressor-exposed individual activates a stress response in other individuals. Stress responses could be activated via perceiving stress in others, as a form of inadvertent social information [29,30] being acquired via indirect cues (e.g. increased vigilance, breathing rates or aggression). Such cues could be useful, for example, for dispersing individuals prospecting new habitats and acquiring information on suitability (e.g. long-term food availability and predation pressure), or by propagating information about threats in the environment not witnessed first-hand. Stressed individuals can also signal a stressor directly (e.g. by alarm calling or releasing a pheromone), thereby potentially inducing an activation of the stress system in others. By contrast, producing signals or cues without activation of a stress system (e.g. deceptive alarm calls as ploys [31], or flying away from a predator without perceiving it as threatening [32]) might induce stress responses in others, but would not be considered as stress transmission. In many cases, stress transmission via cues or signals are likely to have qualitatively similar outcomes, with differences in transmission modes (e.g. one-to-one versus one-to-many) having a greater impact than differences in mechanisms (e.g. indirect cues versus signals) [33]. For example, heart rate matching [34] or signalling a threat to a social partner could have more similar group-level outcomes (e.g. the size of an outbreak of stress activation) than releasing pheromones to a whole group. So far, most in-depth research on stress transmission has been conducted in humans, alongside studies on a limited number of other species. Given that pathways of the stress response are evolutionarily deeply rooted and structurally highly conserved among vertebrates [35] (albeit with some functional differences [36]), stress transmission could be a common, and largely under-acknowledged, process across social animal taxa.

In this paper, we first review examples of stress transmission and consider its potential to operate as means of social information transmission. We then discuss stress transmission through the lens of animal collectives, defined as any set of animals where the actions of one or more individuals impact the behaviour of others. Specifically, we propose that individual variation, group size and composition, and social relationships can shape stress transmission, and that stress transmission can impact emergent properties of collectives. We conclude by suggesting research approaches to advance this underexplored phenomenon and generate new perspectives on the dynamics of stress in animal collectives.

## 2. The social transmission of stress in humans and animals

Four decades of research on transmission of physiological stress among humans have established that responses to stressors exhibited by one person can be transmitted to another [28,37]. For instance, emotional reaction to stressors experienced in the work environment can propagate to partners, family and close friends, transmitting through social networks similarly to pathogens [38]. Although stronger social bonds are more receptive to transmission [39], stress responses can also be transmitted along weaker social links and among individuals unfamiliar with each other. One study found that the magnitude of cortisol increase depended upon emotional closeness, and whether observers were physically close or watched the stress induction through a screen from afar [40]. Stress transmission is thought to help individuals navigate their dynamic social environments, allowing them to understand what others are experiencing [41]. However, social stress transmission can also cause individuals to experience detrimental forms of stress system activation—seen for example in chronic stress (box 2)—without experiencing traumatic events themselves [28].

While studies in humans have been instrumental in understanding stress transmission, we know remarkably little about whether, when and how activation of the stress system can spread among non-human animals. Most existing knowledge comes from laboratory experiments with rodents. Adult rats housed with a partner that experienced daily defeat stress from aggressive individuals—a common experimental paradigm in rodent studies—express behavioural and physiological changes corresponding to those of their stressed partner, including similar social avoidance behaviour, cardiac autonomic activation and upregulation of stress-associated hormones [42]. Studies of social transmission of defeat stress have the potential to reveal more insights into the neurobiological mechanisms of stress transmission [43]. For example, laboratory mice either directly exposed to a stressor (footshock) or to an individual previously exposed to a stressor underwent the same synaptic changes, revealing neural effects [44]. Together, these studies confirm the potential for stress transmission in non-human vertebrates and show that the impacts of transmitted stress are similar to those of direct exposure to stressors, albeit evidence remains limited in terms of taxonomic and contextual breadth.

Experimentally induced stress transmission has also been observed in wild animals. In yellow-legged gulls (*Larus michahellis*), both chicks implanted with the stress-associated hormone corticosterone and their nest-mates expressed faster crouching and hiding in response to adult alarm calls [45]. While making them safer from predators, such reactions come at high costs. At fledging age, implanted chicks and their nest-mates were smaller, had fragile plumage and showed oxidative damage, aligning with the costs of chronic stress [46]. The benefits of the stress response—mobilizing energy reserves and putting the body in a high alert state when facing a potentially lethal threat (increased survival)—often outweigh the costs arising from repeated or longer term exposure (suppression of growth, repair, immune functions, reproduction and putting strain on the cardiovascular system, e.g. [46]; box 1). Yet, whether an individual that never encounters the stressor first-hand can also gain benefits warrants further investigation, and additional studies in wild and semi-natural settings are needed.

Physiological interactions do not necessarily entail the activation of stress responses in others, with social bonds often facilitating downregulation following exposure to stressors [47]—a process that is known as social buffering (reviewed in [48,49]). In humans, the impact of stressful situations can be attenuated by the presence of pleasant social company, and stressful situations even trigger a higher need for social company, which appears to be linked to the oxytocin system [50]. In birds, domestic hens (*Gallus gallus domesticus*) reduce behavioural stress responses of their chicks to being startled by air puffs through their mere presence [51]. In a range of species, support from social partners, particularly those deemed to be reliable associates, reduces the frequency and/or intensity of stress responses (reviewed in [52]), presumably by reducing neural activation of the stress system [47]. However, in rodents, individuals that serve as a social buffer for others can experience an activation of their own stress axis [53], thereby limiting subsequent buffering capacity [54]. In addition to buffering the stress response of conspecifics via social support, some individuals might be insensitive to a given stressor, thereby blocking social stress transmission and preventing further social amplification. Understanding in which contexts stress responses are buffered, or transmission is blocked, and finding potential links or differences between the underlying mechanisms of these processes, are important directions to follow to promote health and well-being of both wild and captive animals.

## 3. Stress transmission and social buffering in animal collectives

**Figure 1.**
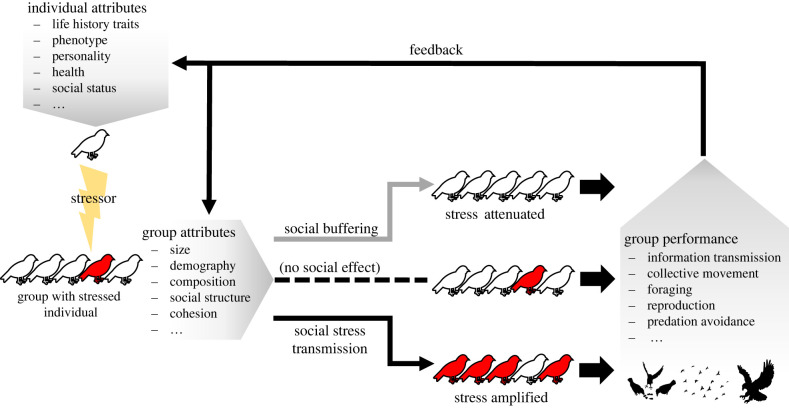
Stress transmission in animal collectives. Individual attributes make members of social groups more or less susceptible to external stressors in their environment by shaping their propensity to encounter stressors and their physiological responses. If the stress response is activated in one individual, changes in its behaviour and physiology can provide cues causing a stress response in conspecifics. Individual and social attributes, or behavioural context, can determine whether a stress response is transmitted (amplified), buffered (attenuated), or if there is no effect. Changes in the composition of the group, or in the interactions among individuals, can alter group performance in collective tasks and other emergent collective properties. The impact on group performance could then feed back onto how individuals interact with their environment, for example by increasing predator alertness or reducing social cohesion, potentially altering their state (e.g. changed reproductive status or health) and shaping their future response to stressors. (Online version in colour.)

There is now sufficient evidence to plausibly predict that transmission of stress is widespread across vertebrates. While mostly focused on stress transmission between dyads of individuals, existing studies provide a valuable starting point, suggesting that stress transmission could be occurring on larger scales. Many animals form tight spatio-temporal associations—such as aggregations, groups or colonies—providing the substrate through which stress responses can be transmitted or be attenuated. One benefit of stress transmission is that an aversive stimulus does not need to be directly experienced by all individuals to elicit a collective response. An individual encountering a predator outside of its colony could lead to other individuals matching its stress response (figure 1). Such ‘eavesdropping’ on the stress response of conspecifics (or even heterospecifics) could represent a very basal and direct form of social information use [55]—acquiring information about the environment from the behaviour of others—without requiring active decision-making (e.g. choosing whether to remain or flee) or by lowering the threshold for making a decision (e.g. to flee). However, the transmission of acute stress responses could potentially be costly, especially if maladaptive mismatches between cue and response occur, or if stress activation becomes chronic. Such outcomes could be plausible under human-induced environmental changes where animals might not be able to assess risks and appropriate responses correctly [15].

One factor that will underpin stress transmission, and/or affect its outcomes, is individual variation. Differences in severity and sensitivity of the stress response have been linked to early-life conditions [56], personality [57], prior experience [58] or genetic predisposition for coping styles [59]. Other behavioural traits, including movement patterns or risk taking, can also determine the propensity to encounter stressors [60]. Whether being susceptible to transmitted stress is shaped by the same traits as those affecting susceptibility to direct stressors, and which traits shape an individual's propensity to transmit stress responses to others, remains to be investigated. Overall, the dynamics introduced by individual variation might help to maintain balance between the hypo- and hypersensitivity to a stressor [61], in the same way that personality composition of groups underpins collective functions [62] and resilience of populations [63].

The potential for stress responses to spread or be contained by the collective could be modulated by other aspects of the social environment, such as the relative size of the pool of affected and unaffected individuals. Insights could be gained from studies of leadership in animal collectives, which have suggested that a small number of informed individuals can lead large numbers of uninformed individuals [64]. This means that an initial response by a few individuals could drive stress-mediated behavioural changes in large groups, with stress transmission potentially causing major consequences (e.g. stampedes [65]). While few individuals could promote widespread activation of the stress system, social contagion theory [66] suggests that more individuals would be necessary to buffer or block transmission. Thus, there are likely to be different rules governing social up- and downregulation of the stress response.

Social structure, like dominance hierarchies or differentiated social relationships, could also underpin physiological interactions. Stress transmission or buffering may not occur equally among all pairs of individuals, with biases similar to those observed with social learning [67]. Like in humans, the quality of social relationships has been demonstrated to impact social buffering [40] in macaques (*Macaca sylvanus*; [68]) and prairie voles (*Microtus ochrogaste*; [53])—even though similar processes have been observed in three-spined sticklebacks (*Gasterosteus aculeatus*) [69], a species lacking strong social bonds. Associating with group members experiencing acute or chronic stress could also lead to alteration of social structure itself [70]. Social cichlid fish (*Neolamprologus pulcher*) temporarily held out of water recovered faster (showing lower cortisol levels and neural transcript abundance) when in their group but experienced a subsequent reduction in social affiliations [47]. Reduced social connectivity could then induce a shift from social buffering to blocking stress transmission after a future encounter with a stressor, or promote transmission by eroding buffering capacity. Changes in social structure have also been linked to reduced group performance [71], which in itself could become a stressor (e.g. not getting sufficient food). Costs associated with social buffering (e.g. elevated susceptibility and loss of affiliations) might promote selectivity in social interactions, such as avoiding very stress-responsive individuals.

The interplay between stress system activation and changes in social structure is likely to be highly dynamic. It may even follow a hysteresis—a path dependency exhibited by many physical and biological systems, where a system's current state depends on its previous state(s). For example, agar melts at 85°C and solidifies at 40°C, meaning that its state at 60°C depends on its past state. Such hysteresis effects are common in nature. For example, individuals can increase sociality when faced with a weak pathogen without immediate increases in virulence, but subsequently decreasing sociality when virulence is high may not immediately reduce virulence [72]. Similar dynamics may arise between buffering capacity and stress transmission [71]: once stress systems are widely activated among individuals, any recovery of the social system may not immediately reintroduce buffering capacity. Initial physiological states of other group members, their past experiences and their resulting responses will thus probably all contribute to shape stress transmission processes in social groups and their outcomes.

A final major question is what form of transmission stress transmission takes. Although previously suggested to spread in similar ways to pathogens [38] (i.e. simple transmission), stress transmission probably has properties more similar to other forms of information transmission. For example, while individuals in a group can activate a stress response by direct exposure to a stressor, akin to a group member ‘innovating’ new information, the transmission of pathogens is usually only possible via contact with an infected individual. This has some major consequences for our understanding of transmission as it could take a variety of forms. For example, it can require overcoming an activation threshold, where enough individuals need to respond (or respond strongly enough) before any transmission takes place. Such ‘complex contagion’ can make groups less susceptible to local fluctuations in the information landscape, as well as less sensitive to noisy cues [73]. The potential for complex contagion to protect groups from costly outcomes, like the amplification of stress responses in the group in situations where it is maladaptive (*sensu* [67]) makes it highly likely that stress transmission in collectives should follow the form of complex contagion. Thus, understanding of this process, and the extent to which stress can percolate through social networks, remains a fundamental gap in knowledge.

## 4. Insights from collective animal behaviour

The study of collective animal behaviour has had major impacts on our understanding of how social interactions drive higher level processes that modulate interactions between organisms and their environment. For example, highly coordinated collective movement can emerge from a combination (or a subset) of three simple local interaction rules—attraction, alignment and avoidance—to produce complex phenomena [74,75]. Emergent properties of collectives include pooling information among individuals that result in accurate collective decisions [76], making collectives inherently better at extracting information from noisy environments [77]. For example, fish schools can track patches of shade without any individual having information about the global distribution of light and dark patches [78]. In the context of stress transmission, similar processes could involve estimating the level of threats and when to select to mount a stress response. The study of animal collective behaviour thus represents a natural and exciting starting point for considering the physiological underpinnings of social interactions and how stress transmission can have consequences for social groups.

Animal collective behaviour could facilitate sensing of the environment via modulation of stress physiology—where the physiological modulation represents a possible additional interaction rule. For example, the process of achieving synchrony in breeding colonies of birds has been attributed to interacting stress levels between breeding pairs, where each breeding pair responds to small changes in agitation of their neighbours to adjust their own physiology and reproductive schedule [79]. Synchronized breeding schedules can provide benefits, such as through safety in numbers for their offspring, and promote future social bonds [80]. Interactions via stress levels might also operate as a quorum-like process, whereby a colony integrates (or pools) information emerging from individuals' experiences of the environment (both physical and social) and breeding is initiated once some threshold is achieved. Such physiologically mediated collective behaviours could benefit individuals by modulating responses to environmental stressors or challenges.

The emergent properties of collective behaviour can be quite sensitive to individual heterogeneity (reviewed in [81]), and the presence of variation in behaviours within a collective has been shown to generate adaptive outcomes [62]. However, stress transmission could reduce heterogeneity in behaviour if individuals conform to the group (as previously shown with differences in hunger states [82]), which could either induce costs (if the collective fails to express important behaviours) or benefits (if the collective increases attention to maintaining group cohesion). Physiology has been suggested to play a key role in orchestrating the social make-up and interactions in animal groups [83]. For example, it can alter the relative drive for individuals to remain cohesive versus to seek new resources as their state of need (e.g. hunger) changes [84]. By changing how individuals interact with one another—and thereby changing the collective's behaviour—stress physiology could represent a major feedback process when coupled with collective behaviour. However, what exactly are the population-level consequences of this process and can stress transmission drive differences in performance across groups or colonies that might outwardly appear to be very similar?

The outcome of stress responses being transmitted and amplified through collectives might drive constraints in the functionality and performance of groups. Individuals that were exposed to stressors or conspecifics with acute stress responses might reduce synchronization, impacting coordination in collective processes. For example, the collective cortisol and testosterone levels in human groups predict their performance in collective tasks [85]. In sticklebacks, swarms containing individuals infected with a parasite that alters movement patterns undergo changes in social interactions, coordination between individuals and leadership patterns [86]. Since acute stress can similarly modulate movement patterns [87] and motor functions [88], it has the potential to have similar effects on collectives. It remains unclear whether there are mechanisms in place within animal groups that provide some (emergent) control over properties of the collective and stress transmission dynamics (e.g. whether stress is transmitted or buffered), with one possibly important mechanism being complex contagion (see previous section).

## 5. Future directions for studying stress transmission in animal collectives

Studying the mechanisms and consequences of stress transmission, and particularly discerning this effect from exposure to common environments, will be challenging. Nevertheless, some studies have been able to overcome these challenges, even outside of controlled laboratory conditions [45]. Doing so requires integrating multiple approaches: experimentally manipulating individuals and measuring the stress levels and responses of their group mates, and correlational studies in the wild for establishing testable hypotheses and understanding fitness consequences. Experimental manipulations will require introducing stressors into social groups without simultaneously triggering stress responses in everyone else (e.g. avoiding the experimenter acting as a stressor). This is particularly challenging because even the act of removing group members could induce stress responses in others [89]. Probably for this reason, studies on stress transmission to date have focused on dyads of individuals and have been conducted in a limited number of species and contexts: dyads of humans [40], laboratory-housed rodents [42,44] and fishes [69], and nests of birds [45]. For a correlational approach, the challenge is to simultaneously quantify acute stress responses or chronic stress among individuals and repeated sampling in ways that avoid disturbing group members, while capturing multi-system responses (e.g. endocrine and behavioural responses) and changes over relevant timescales for stress transmission [90].

Owing to the ease of measuring endocrinological responses (e.g. compared to responses of the sympathetic nervous system), these are often used in stress-related studies. Such hormonal measures can be collected using indirect sampling methods (e.g. faecal sampling [91]), although they often lack the temporal resolution to identify the initial source of stress and subsequent pathways of transmission. A challenge with hormonal stress assessment is the underlying individual variation and fluctuations in glucocorticoid levels (e.g. [92]). Further, no single measure gives a definite answer on the ‘stress’ that an individual is experiencing [93], and a set of physiological indicators may need to be exploited along with behavioural and performance measures. Frameworks for the integration of stress across multiple systems (sympathetic nervous system, hypothalamic-pituitary-adrenal-axis and psychological/behavioural responses) have already been recommended for stress assessment in humans [94] and may in a similar fashion be adapted for animal research. Non-experimental studies might also struggle to determine whether stress responses were transmitted or whether stress levels in the group arise owing to the correlated experience among group members (e.g. their simultaneous exposure to a predator). Thus, researchers will be challenged to find creative and innovative approaches to overcome these difficulties and expand the research on stress transmission to a wider range of species and different group sizes and compositions.

We suggest pursuing three guiding questions: (i) when and where stress transmission occurs, (ii) the consequences of stress transmission in social groups, and (iii) the prevalence of stress transmission in wild populations. Apart from the challenge of quantifying and manipulating stress levels of individuals, understanding transmission patterns in social groups will require good knowledge of the social relationships and encounters among individuals. This is now possible with modern tracking technologies in both laboratory (e.g. automated camera tracking [95]) and wild (e.g. using passive integrated transponders [96], camera traps [97] or deep learning-based methods [98]) settings. Fine-scale tracking will be important for determining whether stress transmission occurs via physiological cues or signals, or is driven by changes in the quality of interactions among individuals (e.g. increased aggression). Analytically, social network analysis [99] is a useful framework for mapping social relationships and transmission patterns, and is widely used to study information and disease transmission. The interplay between individuals, their traits and how networks shape stress transmission patterns would be best quantified by integrating the susceptible-infected-recovered models used for studying disease transmission (e.g. [100]) with network-based diffusion models that are used to model information transmission [101–103]. Such an approach could be effectively used to answer whether, when and how stress transmission occurs (question (i)).

Traditional observational methods and behavioural paradigms will also be important for quantifying consequences of stress transmission. Group-level performance measures that could be studied in response to stressors include foraging efficiency [71], reproductive success [104], synchronization of breeding [105] and coordination of movement [106]. These represent clear contexts where having some proportion of the group affected by stress transmission could have consequences on the performance of the whole group (question (ii)). Commercial animal husbandry is one domain where social dynamics have been linked to animal welfare considerations and productivity [107,108], and these could further benefit from considering the impact only a few stressed individuals could have on the rest of the group. For experimental studies, individual stress responses and glucocorticoid levels can be altered experimentally, by exposing animals to alternating psychological stressors [109] or exogenous glucocorticoid administration [110]. Where we cannot manipulate stress levels of individuals (behaviourally or endocrinologically), owing to ethical, logistical or practical limitations, correlational studies can also provide important insights. One study showed the synchronization of glucocorticoid levels between dogs and their owners [111]. Similar studies will be important when testing for evidence of stress transmission processes in wild animals (question (iii)).

Field-deployable tracking technologies, ranging from GPS tags to heart rate loggers, have also rapidly advanced over recent years. These allow researchers to simultaneous collect large quantities of data on movement [112] and physiological parameters [113], making it possible to study individuals and collectives over time and across contexts. Importantly, longer term tracking facilitates within-individual comparisons following different ecological stimuli, or when exposed to the same ecological stimulus but in different social environments. This could help identify variation in stress transmission, for example between taxa, ecological niches and conditions, some of which might be reflected and measurable in different parameters of collectives, like their size and density of social interactions.

As in all animal research, studies on stress transmission must observe the highest standards of animal ethics and well-being. Studies must be carefully planned, applying the 3R (replace, reduce and refine) methods to improve animal welfare in research, and with the balanced appraisal of knowledge gain and invasiveness. A major practical goal in understanding stress transmission in animal groups is to improve conservation efforts, for instance predicting the health of populations that might encounter disturbance and improving animal experimental and husbandry practices.

## 6. Conclusion

Stress transmission is an impactful, yet largely overlooked, process that could be common in social vertebrates. Animal social dynamics can be complex and span behavioural contexts, making disturbances particularly profound and far-reaching. Stress transmission processes can cause and propagate such disturbances in social groups and modulate the emergent properties of animal collectives. However, more insights from research of animal social systems are needed to unravel the mechanisms and the consequences of stress transmission. These will not only be of theoretical interest but prove highly relevant for applied research. Mapping how stress spreads along animal social ties, for instance, can improve commercial animal husbandry conditions and help minimize the impacts of anthropogenic stressors on wild populations.

## Data Availability

No data were used for this article.
